# Immobilization of *Pseudomonas* sp. DG17 onto sodium alginate–attapulgite–calcium carbonate

**DOI:** 10.1080/13102818.2014.961123

**Published:** 2014-11-07

**Authors:** Hong Qi Wang, Fei Hua, Yi Cun Zhao, Yi Li, Xuan Wang

**Affiliations:** ^a^ College of Water Sciences, Beijing Normal University, Beijing100875, China

**Keywords:** *Pseudomonas*, crude oil, immobilization, carrier, biodegradation

## Abstract

A strain of *Pseudomonas* sp. DG17, capable of degrading crude oil, was immobilized in sodium alginate–attapulgite–calcium carbonate for biodegradation of crude oil contaminated soil. In this work, proportion of independent variables, the laboratory immobilization parameters, the micromorphology and internal structure of the immobilized granule, as well as the crude oil biodegradation by sodium alginate–attapulgite–calcium carbonate immobilized cells and sodium alginate–attapulgite immobilized cells were studied to build the optimal immobilization carrier and granule-forming method. The results showed that the optimal concentrations of sodium alginate–attapulgite–calcium carbonate and calcium chloride were 2.5%–3.5%, 0.5%–1%, 3%–7% and 2%–4%, respectively. Meanwhile, the optimal bath temperature, embedding cell amount, reaction time and multiplication time were 50–60 °C, 2%, 18 h and 48 h, respectively. Moreover, biodegradation was enhanced by immobilized cells with a total petroleum hydrocarbon removal ranging from 33.56% ± 3.84% to 56.82% ± 3.26% after 20 days. The SEM results indicated that adding calcium carbonate was helpful to form internal honeycomb-like pores in the immobilized granules.

## Introduction

Bioremediation, the use of microorganisms or microbial processes to degrade environmental contaminants, is considered to be a most cost-effective technique to clean up ground water, soils, lagoons, sludge and process-waste streams.[1] Once applied, the success of a bioremediation procedure depends on the establishment of microorganisms and on the expression of the necessary degradative genes.[2] However, the use of free cells for the degradation of various toxic compounds has a number of disadvantages like their low mechanical strength, low density of cells and difficulty in biomass effluent separation.[3] In this case, the use of inoculant microorganisms with a carrier material, which should be non-toxic, should have a constant quality and is cost effective, is an attractive option.[4–6]

The advantages of immobilized technique include easy separation of the cells from the carrier material, reusability, greater productivity due to high cell concentrations achieved and the protection of cells against harsh environmental conditions.[7–11] For these properties, using immobilized cells improved the biodegradation of petroleum-contaminated soil.[12,13] Studies have shown that polymer types play a dominant role in determining the properties of the microspheres. Examples of synthetic gels include poly(carbamoyl)sulphonate, polyhydroxyethylmethacrylate (polyHEMA) and polyacrylamideand polyvinyl alcohol (PVA).[14] Microspheres are almost exclusively produced using water-soluble polymers which provide a high degree of permeability for nutrient sand metabolites with a low molecular weight, thus providing optimal conditions for the functioning of immobilized microbial cells. Sometimes, the biodegradability of indigenous microorganisms is unstable, because of the complex soil environment. In this case, the carrier material needs to be studied further. Two categories of hydrogel material, natural and synthetic, are used for cell immobilization.[15] As for the former agar, agarose, polyacrylamides, carrageen and alginate are common examples.[4] Alginate–chitosan–alginate was used to immobilize *Saccharomyces cerevisiae* culture and parameters, such as microcapsules core state, initial cell density, microcapsule diameter and membrane formation times, were monitored.[16] Similarly, calcium alginate beads were used in the immobilization of bacteria culture and immobilization parameters that can influence the viability of bacteria have been optimized, such as alginate concentration, calcium chloride concentration and hardening time of gel beads in calcium chloride.[17]

In this work, a new carrier material composed of sodium alginate, attapulgite and calcium carbonate, used to immobilize *Pseudomonas* sp. DG17, was constructed. The proportions of different components and the construction laboratory parameters, including bath temperature, embedding cell amount, reaction time and multiplication time, were investigated. The results of this study could provide a new cost-effective immobilization carrier material for the bioremediation of petroleum-polluted soil.

## Materials and methods

### Bacterial and growth condition

*Pseudomonas* sp. DG17 (CGMCC ID: 5052; GenBank ID: JN 216878) was isolated from petroleum-contaminated soils by an enrichment culture technique using crude oil as the sole source of carbon and energy. The culture was maintained at 4 °C on crude oil medium and transferred monthly.[18]

For cell proliferation, *Pseudomonas* sp. DG17 was inoculated into LB medium (5% yeast extract, 10% peptone and 5% sodium chloride, pH 7.0) at 10 ºC for 48 h. For crude oil biodegradation, the mineral salt medium (MSM) contains (per litre) 0.4 g Na_2_HPO_4_, 0.15 g KH_2_PO_4_, 0.1 g NH_4_Cl, 0.05 g MgSO_4_·7H_2_O, 0.0015 g CaCl_2_, 0.1 g NaNO_3_ and 1 mL trace medium (per 100 mL solution containing 0.5 mg CuSO_4_·5H_2_O, 1.0 mg H_3_BO_3_, 1.0 mg MnSO_4_·5H_2_O and 7.0 mg ZnSO_4_). The pH of the medium was adjusted to 7.0 and sterilized by autoclaving at 121 °C for 20 min. The sterilized medium was then supplemented with immobilized cells of DG17. The medium used for pre-cultivation of the strain contains (per litre) 20 g glucose, 3 g yeast extract, 2 g peptone, 1 g NH_4_NO_3_, 0.2 g MgSO_4_·7H_2_O and 0.2 g KCl. The pH of the medium was adjusted to 7.0 and sterilized by autoclaving at 121 °C for 20 min.

Clean soil samples were collected from a farmland field nearby Baiwangshan of Beijing. The basic physical and chemical properties of soil samples are listed in Table 1. Crude oil contaminated soil samples were collected from Huabei Oilfield. The basic physical and chemical properties of soil samples are listed in Table 2. Crude oil was bought from Huabei Oilfield in China.

**Table 1.** Physical and chemical properties of clean soil samples.

Element	Total C	Total H	Total N	Total P	Total K
Content (mg/g)	14.25	4.47	0.64	0.481	19.593

**Table 2.** Physical and chemical properties of crude oil contaminated soil samples.

Element	Total C	Total H	Total N	Total P	Total K
Content (mg/g)	105.4	15.86	0.52	0.506	14.824

### Immobilized carrier of *Pseudomonas* sp. DG17

To study the proportion of carrier, a 4^3^ design was implemented. As shown in Table 3, the independent variables studied were sodium alginate, attapulgite, calcium carbonate and calcium chloride. The activity of succinodehydrogenase was tested to assess the immobilized microbial activity. During the experiment, water temperature was controlled at 50 °C, the embedding cell amount was 10% (v:v), the reaction time was 24 h and the pre-cultivation time was 48 h.

**Table 3.** Orthogonal test design of four independent variables.

	Independent variables (%, w:w)			
Levels	Sodiumalginate	Attapulgite	Calcium carbonate	Calcium chloride
1	2.5	0.5	3	2
2	3	0.75	5	3
3	3.5	1.0	7	4

### Laboratory immobilization parameters of *Pseudomonas* sp. DG17

To study the influence of different parameters on the succinodehydrogenase activity of immobilized *Pseudomonas* sp. DG17 cells, bath temperature, embedding cell amount, reaction time and multiplication time were varied.

The bath temperature was controlled at 30, 40, 50, 60, 70 and 80 °C. The embedding cell amount was 1% (v:v), the reaction time was 24 h and the pre-cultivation time was 48 h. To study the embedding cell amount on the succinodehydrogenase activity of immobilized cells, the initial cell amount was 1%, 2%, 3%, 4% and 5%, respectively. The bath temperature was 50 °C, the reaction time was 24 h and the pre-cultivation time was 48 h. The reaction time was 6,12, 18, 24 and 30 h. The bath temperature was 50 °C, the embedding cell amount was 4% and the pre-cultivation time was 48 h. The pre-cultivation time was 12, 24, 48, 60 and 72 h. The bath temperature was 50 °C, the embedding cell amount was 4% and the reaction time was 24 h.

### Scanning electronic microscopy of immobilized granule

Samples of immobilized cells were collected and washed with 0.1 mol L^−1^ phosphate buffer (pH 7.2), fixated with 2.5% glutaraldehyde, washed with 0.1 mol L^−1^ phosphate buffer pH 7.4 and post-fixed in 1% osmium tetroxide for 2 h. After several washes in the same buffer, the cells were dehydrated through a partial ethanol dehydration series (50%, 70%, 85%, 95% and 100% (V:V) ethanol) to preserve the hydrophilic and hydrophobic interfaces. The samples were critical point dried (HCP-2, Hitachi, Tokyo, Japan) and sputter-coated with ion coater instrument (IB-3, Eiko, Japan) in argon atmosphere to an approximate thickness of 10–20 nm, and examined by a scanning electron micrograph (S-4800, Hitachi, Tokyo, Japan).

### Crude oil biodegradation by immobilized cells

The flasks contained 10 g of polluted soil. Before the experiment started, immobilized cells of *Pseudomonas* sp. DG17 and MSM were added into the flasks. After that, the flasks were sealed by sealing membrane and incubated at 10 °C. After incubation for 20 days, the soil was freeze-dried at −20 °C to remove the water and the residue crude oil in the soil was analysed. The control group that did not contain immobilized cells of DG17 was also examined. Three different samples were conducted simultaneously for standard deviation analysis.

To study the influence of different parameters on the crude oil biodegradation by immobilized cells of *Pseudomonas* sp. DG17, substrate concentration, immobilized cell amount and different ratio between water and soil were varied according to the experimental design. The crude oil concentrations were 100,600, 58,790 and 1000 mg kg^−1^. The initial cell amount was 10% and the ratio between water and soil was 2:1. The initial cell amount was 2.5%, 5% and 10%, respectively. The crude oil concentration was controlled at 6000 mg kg^−1^. The ratio between water and soil was 1:2, 1:2 and 1:3, respectively. The crude oil concentration was controlled at 6000 mg kg^−1^ and the cell amount was 5%.

### Cell immobilization

Sodium alginate and attapulgite were both dissolved in distilled water and sterilized by autoclaving at 120 °C for 30 min. After the temperature of the solution declined to 40–50 °C, calcium carbonate was added into the solution to form embedding medium. For cell proliferation, *Pseudomonas* sp. DG17 was inoculated into LB medium at 10 ºC for 48 h. After that, the LB medium that contained the suspended cells of DG17 and embedding medium (1:10, v:v) was transferred into 4% of calcium chloride solution by a constant flow pump to form immobilized granules. Then, the immobilized granules were collected, transferred into flasks containing HCl to remove calcium carbonate, immobilized at 4 °C for 24 h and transferred into proliferation medium for 48 h. Finally, the immobilized granules were collected and washed by distilled water for three times.

### Analysis methods

#### Immobilized microorganism relative activity

The succinodehydrogenase activity was considered as a standard to assess the microorganism activity. In the studies, the ratio between the succinodehydrogenase activity of immobilized cells and that of suspended cells was considered to represent the relative activity of the immobilized cells. In this case, cells which were embedded in the carrier should be freed. One millilitre of immobilized cell granule was dissolved in 9 mL of 0.2 mol L^−1^ of sodium citrate solution. After that, 30 uL of the solution was placed into a colorimetric tube, diluted 100 times and 300 uL of 0.1 mol L^−1^ methyl thiazolyl tetrazolium were added. The colorimetric tube was transferred into the water bath warmed and kept at 30 °C for two hours. Freed immobilized cells were collected at 10,000 rpm and transferred into 4 mL of dimethyl sulphoxide. Finally, the relative enzyme activity was tested at OD_525_.

### Total petroleum hydrocarbon of soil

The total petroleum hydrocarbon (TPH) of soil was tested by accelerated solvent extraction. Ten grams of soil that has been freeze-dried was placed into the extraction pool and extracted by hexane and acetone (1:1, v:v) two times. After that, the liquid in the receiving flask was removed by nitrogen flushing. When the weight of receiving flask did not change any more, the weight of the residual crude oil was recorded as *G*_oil_. Meanwhile, the control group that was not supplemented with cells was used for a biotic loss analysis of crude oil, and the crude oil content in the soil was calculated as *K*_oil_/*K*_soil_. The crude oil content in the soil was calculated as *G*_oil_/*G*_soil_. The biodegradability of TPH was calculated as (1 − *G*_oil_/*G*_soil_/(*K*_oil_/*K*_soil_)).

## Results and discussion

### Immobilization carrier proportion of *Pseudomonas* sp. DG17

**Figure 1.**
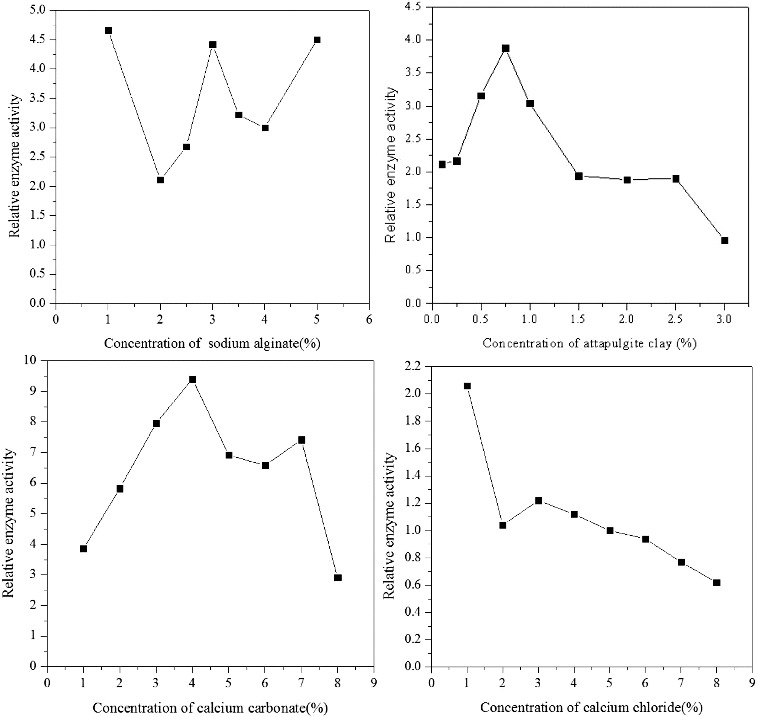
Effect of independent variables on the relative enzyme activity of immobilized cells.

The optimal content of attapulgite was 0.75% (Figure 1). Moreover, when the content of attapulgite was higher than 2%, precipitate occurred during the process of forming sodium alginate–attapulgite gelatin. Meanwhile, when the content of sodium alginate was higher than 4%, the viscosity of sodium alginate–attapulgite gelatin improved which increased the difficulty to form granule. In this case, the content of attapulgite should be maintained between 0.25% and 2%, and the concentration of sodium alginate was controlled at 2.5%–4%.

The optimal relative activity of the microorganisms was achieved when the concentration of calcium carbonate was ranged between 3% and 7% (Figure 1). Moreover, when the concentration of calcium chloride was higher than 4%, the relative activity of the microorganisms declined due to the dehydration of the cells. In our study, the optimal concentration of calcium chloride was ranged between 3% and 7%.

Immobilized cells have higher activity, higher cell density and longer stability than free cells. Hence, the immobilized cells have the potential to degrade toxic chemicals at higher concentration as compared to the freely suspended cells. Meanwhile, an effective and safe carrier for soil-applied microorganisms should be non-toxic and non-polluting, consistent in quality, have a long shelf life, allow sufficient cell activity and cell density and permit accurate release of bacteria into the target site.[19,20] In general, non-toxic natural polymers are recommended for use in soil. Synthetic polymers, for example polyacrylamide, must be handled carefully due to the toxicity of the pre-polymer, and the stability of the polyacrylamide beads in soil. Therefore, they may not be environmentally acceptable.[21] Among gel carriers, polyvinyl alcohol cryogels (PVA) are promising matrices for whole cell immobilization. PVA itself is a biologically compatible, non-toxic, readily available low-cost polymer.[22] For example, the hydrophobized PVA cryogel granules (granule volume 5 μl) contained a sufficient number (6.5 × 10^3^) of viable bacterial cells per granule, all located at the border in water–hydrocarbon systems and allowed high contact of immobilized biocatalyst cells with a hydrophobic substrate and the water phase, providing bacterial cells with mineral and organic nutrients.[23] For diesel-contaminated soil, a ratio of 25 mL PVA (20% w/v) to 15 mL re-suspended cells produced a gel that had the necessary consistency. This represented a final PVA concentration of 12.5%. The advantage of co-immobilization when PVA is used to encapsulate bacteria is that the absorbent has better contact with the oil, which leads to higher oil content in the vicinity of the hydrophilic but macroporous matrix.[24] However, the procedure for cell immobilization within PVA is not a simple process compared to that used for the entrapment of biomass into alginate or carrageenan matrices since it is required to freeze and thaw the cells suspended in PVA solution. Sometimes poor results with cryo-PVAG carriers can be obtained particularly if the nature of the PVA cryotropic gelation processes and its accompanying effects are not well understood.[22] According to Becerra et al.,[25] β-galactosidase activity per unit of cell biomass was higher in alginate immobilized than in free-growing cells in the same medium. Similarly, as for *Pseudomonas fluorescens*, cells encapsulated in dried beads or fresh beads survived better than free cells when added to soil. Cells in moist and dried alginate beads also survived a dry/wet cycle in soil, whereas free cells were sensitive to moisture fluctuations.[26] In this case, alginate was a safe and effective material for introducing bacteria into the soil. The carrier was biodegradable, non-toxic and could protect the cells from stress.[27] Moreover, the procedure of immobilizing free cells with sodium alginate–attapulgite was simple compared to that with PVA gel.

### Laboratory immobilization parameters of *Pseudomonas* sp. DG17

The relative activity declined when the bath temperature was higher than 70 °C (Table 4). Meanwhile, it was found that the viscosity of sodium alginate–attapulgite gelatin was high which increased the difficulty to form granules when the temperature was lower than 30 °C. Thus, during the immobilization process, the bath temperature ranged from 50 to 60 °C. Suspended cell amount could also influence the activity of immobilized cells. In our study, when the concentration of initial embedding cells was higher than 2%, the activity of immobilized cells did not increase. Thus, concentration of the initial cells to be embedded was controlled at 2%.

**Table 4.** Different parameters on the succinodehydrogenase activity of immobilized cells of *Pseudomonas* sp. DG17.

Temperature (°C)	OD_525_	Embedding bacteria amount (%)	OD_525_	Reaction time (h)	OD_525_	Multiplication time (h)	OD_525_
30	0.0495	1	0.0322	6	0.0184	12	0.0023
40	0.0366	2	0.0504	12	0.0162	24	0.051
50	0.0482	3	0.0476	18	0.0193	48	0.057
60	0.0532	4	0.051	24	0.0067	60	0.0674
70	0.0176	5	0.0544	30	0.0058	72	0.0668

As for the sodium alginate–attapulgite–calcium carbonate system, reaction time could also influence the activity of immobilized cells. The mechanical strength of immobilized granules was not enough to support the use of immobilized cells when the reaction time was 6 h. Meanwhile, it was found that the activity of cells declined when the reaction time was longer than 18 h. In this case, the optimal reaction time was controlled at 18 h. On the other hand, it was found that the activity of immobilized cells increased along with the multiplication time. However, in the experiment, the activity of sodium alginate–attapulgite–calcium carbonate immobilized cells declined when the multiplication time was longer than 60 h. Thus, in our study, the multiplication time was controlled at 48 h.

In the research of Wiesel et al.,[28] the biodegradability of cells with immobilization material composed of granular clay and slag of lava were studied. It was found that immobilized mixed bacterial culture could completely biodegrade phenol, naphthalene and phenanthrene after incubation for 1, 2 and 15 days, respectively. Furthermore, the immobilized cells showed stable growth and, compared to freely suspended cells, the same degradation sequence as well as an equivalent degradation potential. As for *Mycoplana* sp. MVMB2, the maximum immobilization capacity at various physiochemical conditions for the alkali pre-treated papaya stem was found to be at 320 min time, pH 6.5, 30 ºC temperature and 18.6 × 10^6^ cells/mL initial concentrations. The performance of immobilized cells in batch reactor showed more than 95% phenanthrene degradation within 72 h, whereas free cells were found to require 120 h.[5]

### Structure observation of immobilized cells granule

**Figure 2.**
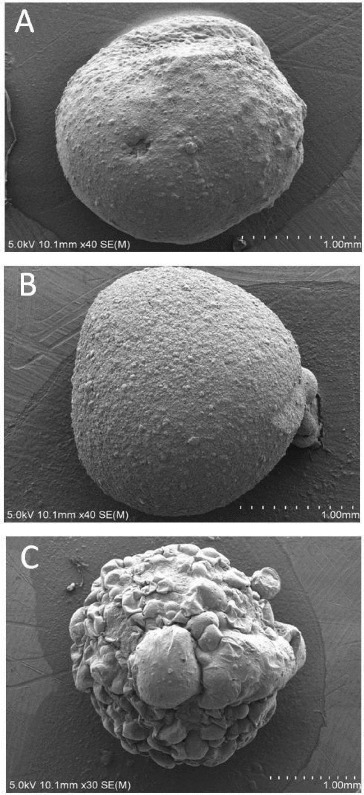
Shape of immobilized granules: (A) sodium alginate immobilized granule; (B) sodium alginate–attapulgite immobilized granule; and (C) sodium alginate–attapulgite–calcium carbonate immobilized granule.

**Figure 3.**
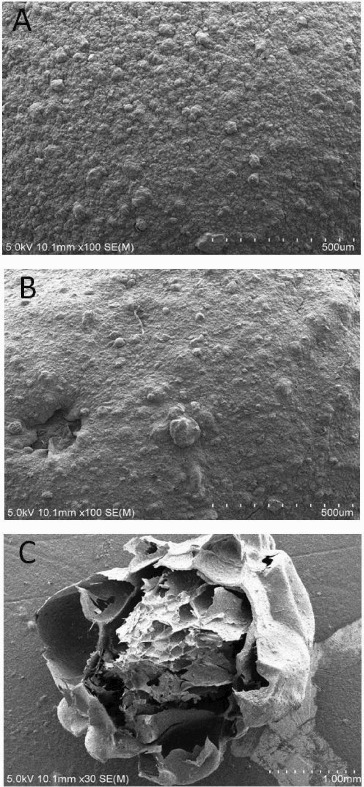
Apparent structure of immobilized granules: (A) sodium alginate immobilized granule; (B) sodium alginate-attapulgite immobilized granule; and (C) sodium alginate–attapulgite–calcium carbonate immobilized granule.

**Figure 4.**
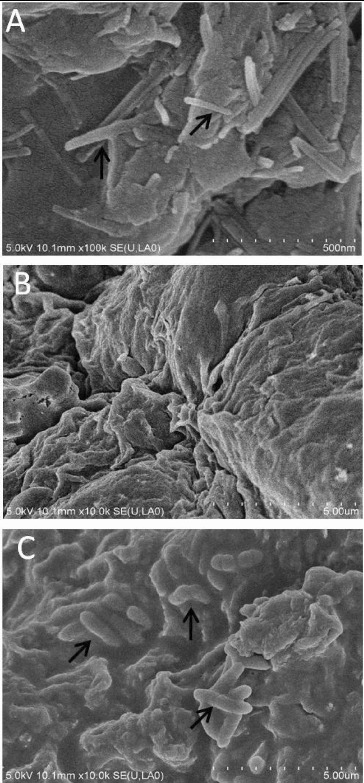
Internal microstructure of sodium alginate–attapulgite–calcium carbonate immobilized granule: (A) the granule did not embed cells of DG17, and the arrows show the attapulgite; (B) the magnify of A; and (C) the granule embedded cells of DG17, and arrows show the immobilized cells of *Pseudomonas* sp. DG17.

The morphology of sodium alginate immobilized granule, sodium alginate–attapulgite immobilized granule and sodium alginate–attapulgite–calcium carbonate immobilized granule is shown in Figures 2 and 3. Compared with the sodium alginate immobilized granule, it was found that adding attapulgite could change the roughness of the granule, which could increase the contact area with crude oil that adsorbed on the soil particles. Meanwhile, the surface of the sodium alginate–attapulgite–calcium carbonate immobilized granule became uneven when adding calcium carbonate which was the optimal carrier for immobilized DG17 cells. Тhe inside of sodium alginate–attapulgite–calcium carbonate immobilized granules has a honey comb texture, and many pore paths exist which was in favour of the free transfer of substrate and nutrients (Figure 4(C)).

Figure 4(A) shows the internal structure of sodium alginate–attapulgite–calcium carbonate immobilized granule with no cells imbedded. The fibre structure is caused by the attapulgite which supported the total skeleton of the granule. Figure 4(B) showed the alveoli-like structure of the granule and the pore paths, which indicated that adding calcium carbonate could provide the mass transfer to support the growth of immobilized cells. Figure 4(C) showed the internal structure of sodium alginate–attapulgite–calcium carbonate immobilized granule that embedded DG17 cells and displayed the distribution of cells on the carrier. Plate counting result showed that 1 mL of carrier embedded 10^8^–10^13^ of DG17.

### Biodegradation of crude oil by immobilized cells of *Pseudomonas* sp. DG17

**Figure 5.**
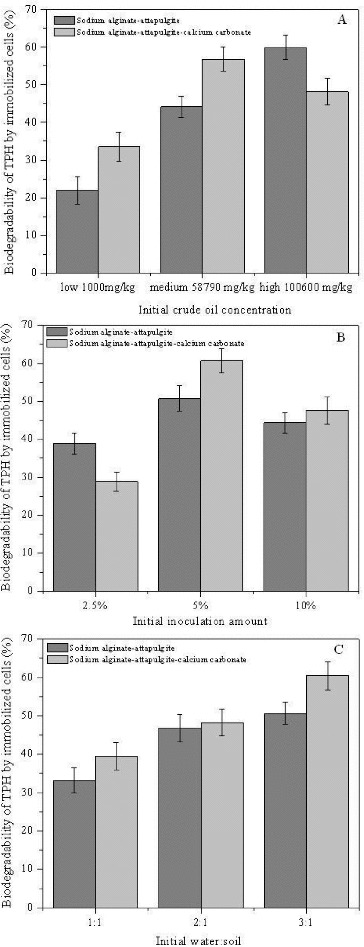
Biodegradation of crude oil by sodium alginate–attapulgite immobilized cells and sodium alginate–attapulgite–calcium carbonate immobilized cells.

After incubation for 20 days, the biodegradability of low, medium and high concentration of crude oil by sodium alginate–attapulgite–calcium carbonate immobilized cells of DG17 was 33.56% ± 3.84%, 56.82% ± 3.26% and 48.13% ± 3.55%, respectively (Figure 5(A)). Meanwhile, the biodegradability of low- and medium-concentrated crude oil by sodium alginate–attapulgite immobilized cells was lower than that by sodium alginate–attapulgite–calcium carbonate immobilized cells which indicated that adding calcium carbonate could improve the activity of cells and accelerate the biodegradation of crude oil in the soil. The sodium alginate–attapulgite–calcium carbonate carrier had a strong resistance to environment factors. When the initial cell amount was controlled at 5% or 10%, the biodegradability of medium- and high-concentrated crude oil by sodium alginate–attapulgite immobilized cells was lower than that by sodium alginate–attapulgite–calcium carbonate immobilized cells (Figure 5(B)). While the biodegradability of crude oil increased along with the ratio between water and soil, the biodegradability of crude oil by sodium alginate–attapulgite immobilized cells was lower than that by sodium alginate–attapulgite–calcium carbonate immobilized cells. When the ratio between water and soil was 3:1, the biodegradability of crude oil by sodium alginate–attapulgite immobilized cells and sodium alginate–attapulgite–calcium carbonate immobilized cells was 60.41% ± 3.61% and 50.66% ± 2.84%, respectively.

Similarly, as for immobilized microorganisms which were supplemented in the aquifer sandy soil,[29] the biodegradability of *n*-alkanes by clay-immobilized cells was greatly improved, and the contents of residue C_12_–C_18_ alkanes were 13.4%–32.3% of the initial alkanes. Studies have shown that free cells of *Arthrobacter citreus* could biodegrade 22 mmol L^−1^ of phenol. However, alginate- and agar-immobilized cells could biodegrade higher concentrations of phenol.[30] When vermiculite was used as immobilized carrier, 95.3% of benzo[a]pyrene was degraded using the co-immobilized system within 42 days, which was remarkably higher than the removal rate of that by the free strains. The immobilized consortium also showed better water stability than the free strains.[31] For the *Pseudomonas* sp. strain, the alginate and polyacrylamide hydrazide immobilized cells were able to degrade phenol at initial concentrations up to 2 g L^−1^ in less than two days, although the free cells did not grow at this concentration.[32] On the other hand, a different immobilization material may have a different effect on the same microorganisms when used for biodegradation of hydrocarbons. For instance, more efficient degradation of naphthalene could be achieved by immobilizing cells in polyurethane foam, rather than in alginate or agar.[33] Moreover, 3% of skim milk or 3% of skim milk and 3% amargosite immobilized microorganisms could biodegrade pollutants well.[34] Similarly, as for the immobilized strain *Rhodococcus coryne bacterioides*, the inoculants formulated with chitin yielded the highest number of cell survival at 4 °C during six months of storage and showed a higher degrading activity of crude oil, compared with the inoculants formulated with chitin and chitosan as carrier materials.[35] Moreover, nutrients are imported during the biodegradation process. According to Alvarez et al.,[36] nutrient addition in silica matrices was studied indicating that the presence of a carbon source without the simultaneous addition of nitrogen was detrimental for immobilized *Escherichia coli*. However, when both carbon and nitrogen sources were present, bacteria were able to survive for longer periods of time. Furthermore, the activity of immobilized cells can be maintained and reused throughout different degradation processes. Thus, the removal efficiency of pollutant was enhanced a lot.[37] The immobilization of strain *Pseudomonas* sp. DG17 has a functional prospective in the crude oil polluted soil treatment.

## Conclusion

In our study, the immobilized carrier proportions of sodium alginate–attapulgite–calcium carbonate were: sodiumalginate 2.5%–3.5%, attapulgite 0.5%–1%, calcium carbonate 3%–7% and calcium chloride 2%–4%. Results of studying the influence of different parameters on the succinodehydrogenase activity of immobilized cells of *Pseudomonas* sp. DG17 during the immobilization process showed that the optimal parameters were as follows: bath temperature 50–60 °C, embedding cell amount 2%, reaction time 18 h and multiplication time 48 h. Under different initial crude oil concentrations, cell amounts and ratios between water and soil, the biodegradability of crude oil in the soil by sodium alginate–attapulgite–calcium carbonate immobilized cells of DG17 was higher than that by sodium alginate–attapulgite immobilized cells. According to the SEM results, the inside of sodium alginate–attapulgite–calcium carbonate immobilized granules has a honey comb texture and many pore paths exist which is helpful to the transfer of substrate and nutrients, thus improved the biodegradation of crude oil in the soil.

## References

[cit0001] Boopathy R (2000). Factors limiting bioremediation technologies. Bioresour Technol.

[cit0002] Providenti MA, Lee H, Trevors JT (1993). Selected factors limiting the microbial degradation of recalcitrant compounds. J Ind Microbiol.

[cit0003] Mulla SI, Talwar MP, Bagewadi ZK, Hoskeri RS, Ninnekar HZ (2013). Enhanced degradation of 2-nitrotoluene by immobilized cells of *Micrococcus* sp. strain SMN-1. Chemosphere.

[cit0004] Leenen ETM, Dossantos VAP, Grolle KC, Tramper J, Wijffels RH (1996). Characteristics of and selection criteria for support materials for cell immobilization in wastewater treatment. Water Res.

[cit0005] Mahalingam BL, Karuppan M, Manickam V (2012). Immobilization of *Mycoplana* sp. MVMB2 isolated from petroleum contaminated soil onto papaya stem (*Carica papaya* L.) and its application on degradation of phenanthrene. Clean-Soil, Air, Water..

[cit0006] Qureshi N, Lai LL, Blaschek HP (2004). Scale-up of a high productivity continuous biofilm reactor to produce butanol by adsorbed cells of *Clostridium beijerinckii*. Food Bioprod Process.

[cit0007] Friel MT, Mcloughlin AJ (1999). Immobilization as a strategy to increase the ecological competence of liquid cultures of *Agaricusbisporus* in pasteurized compost. FEMS Microbiol Ecol.

[cit0008] Park JK, Chang HN (2000). Microencapsulation of microbial cells. Biotechnol Adv.

[cit0009] Rathoreet S, Desai MP, Liew VC, Lai WC, Paul WSH (2013). Microencapsulation of microbial cells. J Food Eng.

[cit0010] Tripathi A, Sami H, Jain SR, Viloria-Cols M, Zhuravleva N, Nilsson G, Jungvid H, Kumar A (2010). Improved bio-catalytic conversion by novel immobilization process using cryogel beads to increase solvent production. Enzyme Microb Technol.

[cit0011] Wang J, Liu P, Qian Y (1997). Biodegradation of phthalic acid esters by immobilized microbial cells. Environ Int.

[cit0012] Quek E, Ting YP, Tan HM (2006). *Rhodococcus* sp. F92 immobilized on polyurethane foam shows ability to degrade various petroleum products. Bioresour Technol.

[cit0013] Xu Y, Lu M (2010). Bioremediation of crude oil-contaminated soil: comparison of different biostimulation and bioaugmentation treatments. J Hazard Mater.

[cit0014] Jen AC, Wake MC, Mikos AG (1996). Review: hydrogels for cell immobilisation. Biotechnol Bioeng.

[cit0015] John RP, Tyagi RD, Brar SK, Surampalli RY, Prevost D (2011). Bioencapsulation of microbial cells for targeted agricultural delivery. Crit Rev Biotechnol.

[cit0016] Qi WT, Yu WT, Xie YB, Ma XJ (2005). Optimization of *Saccharomyces cerevisiae* culture in alginate-chitosan-alginate microcapsule. Biochem Eng J.

[cit0017] Chandramouli V, Kailasapathy K, Peiris P, Jones MJ (2004). Bioremediation of diesel contaminated soil by microorganisms immobilised in a polyvinyl alcohol cryogel. Microbiol Methods.

[cit0018] Hua F, Wang H (2011). Uptake modes of octadecane by *Pseudomonas* sp. DG17 and synthesis of biosurfactant. J Appl Microbiol.

[cit0019] Cassidy MB, Lee H, Trevors JT (1996). Environmental applications of immobilized microbial cells: a review. J Ind Microbiol Biotechnol.

[cit0020] Mcloughlin AJ (1994). Controlled release of immobilized cells as a strategy to regulate ecological competence of inocula. Adv Biochem Eng Biotechnol.

[cit0021] Trevors JT, Van Elsas JD, Lee H, Van Overbeek LS (1992). Use of alginate and other carriers for encapsulation of microbial cells for use in soil. Microb Releases.

[cit0022] Lozinsky VI, Plieva FM (1998). Poly (vinyl alcohol) cryogens employed as matrices for cell immobilization. 3. Overview of recent research and developments. Enzyme Microb Technol.

[cit0023] Kuyukina MS, Ivshina IB, Gavrin AY, Podorozhko EA, Lozinsky VI, Jeffree CE, Philp JC (2006). Immobilization of hydrocarbon oxidizing bacteria in poly(vinyl alcohol) cryogels hydrophobized using a biosurfactant. J Microbiol Methods.

[cit0024] Cunningham CJ, Ivshina IB, Lozinsky VI, Kuyukina MS, Philp JC (2004). Bioremediation of diesel-contaminated soil by microorganisms immobilised in a polyvinyl alcohol cryogel. Int Biodeterior Biodegradation.

[cit0025] Becerra M, Baroli B, Fadda AM, Blanco Mendez J, Gonzalez Siso MI (2001). Lactose bioconversion by calcium-alginate immobilization of *Kluyveromyceslactis* cells. Enzyme Microb Technol.

[cit0026] Trevors JT, Van Elsas JD, Lee H, Wolters AC (1993). Survival of alginate-encapsulated *Pseudomonas fluorescens* cells in soil. Appl Microbiol Biotechnol.

[cit0027] Bashan Y (1986). Alginate beads as synthetic inoculation carriers for slow release of bacteria that affect plant growth. Appl Environ Microbiol.

[cit0028] Wiesel I, Wiibker SM, Rehm HJ (1993). Degradation of polycyclic aromatic hydrocarbons by an immobilized mixed bacterial culture. Appl Microbiol Biotechnol.

[cit0029] Omar SH, Rehm HJ (1988). Degradation of n-alkanes by *Candida parapsilosis* and *Penicilliumfrequentans* immobilized on granular clay and aquifer sand. Appl Microbiol Biotechnol.

[cit0030] Chandrakant K, Aravind M, Manjunath N, Dae JY (2006). Phenol degradation by immobilized cells of *Arthrobactercitreus*. Biodegradation.

[cit0031] Su D, Li PJ, Frank S, Xiong XZ (2006). Biodegradation of benzo[a]pyrene in soil by *Mucor* sp. SF06 and *Bacillus*sp. SB02 co-immobilized on vermiculite. J Environ Sci.

[cit0032] Bettmann H, Rehm HJ (1984). Degradation of phenol by polymer entrapped microorganisms. Appl Microbiol Biotechnol.

[cit0033] Manohar S, Kim CK, Karegoudar TB (2001). Enhanced degradation of naphthalene by immobilization of *Pseudomonas* sp. strain NGK1 in polyurethane foam. Appl Microbiol Biotechnol.

[cit0034] Van Elsaset JD, Trevors JT, Jain D, Wolters AC (1992). Survival of, and root colonization by, alginate encapsulated *Pseudomonas fluorescens* cells following introduction into soil. Biol Fertil Soils.

[cit0035] Gentili AR, Cubitto MA, Marcela F, Rodriguez MS (2006). Bioremediation of crude oil polluted seawater by a hydrocarbon-degrading bacterial strain immobilized on chitin and chitosan flakes. Int Biodeterior Biodegradation.

[cit0036] Alvarez GS, Foglia ML, Copello GJ, Desimone MF, Diaz LE (2009). Effect of various parameters on viability and growth of bacteria immobilized in sol–gel-derived silica matrices. Appl Microbiol Biotechnol.

[cit0037] Chen CY, Chen SC, Fingas M, Kao CM (2010). Biodegradation of propionitrile by *Klebsiellaoxytoca* immobilized in alginate and cellulose triacetate gel. J Hazard Mater.

